# Preparing for the rapid research response to the possible vertical transmission of Oropouche virus: lessons from a decade of congenital Zika research

**DOI:** 10.1016/S1473-3099(24)00618-2

**Published:** 2024-09-27

**Authors:** Elizabeth B Brickley, Demócrito de Barros Miranda-Filho, Ricardo Arraes de Alencar Ximenes, Members of Microcephaly Epidemic Research Group (MERG) Zika Brazilian Cohorts Consortium (ZBC-Consortium), and the LIFE Zika (Long-term Impacts for Families affected by the Epidemic of Zika) Study, Maria de Fátima PM de Albuquerque, Maria do Socorro Veloso de Albuquerque, Maria das Graças Costa Alecrim, Bethânia de Araujo Almeida, Melania Maria Ramos de Amorim, Thalia Velho Barreto de Araújo, Mauricio Lima Barreto, Alessandra Mertens Brainer, Patrícia Brasil, Elizabeth B Brickley, Maria Durce Costa Gomes Carvalho, Marcia da Costa Castilho, Bernadete Perez Coelho, Fanny Cortes, Antonio Ledo A Cunha, Geraldo Duarte, Sophie H Eickmann, Flor Ernestina Martinez Espinosa, Cássia Fernanda Estofolete, Juliana Fontes, Maria Maia Vieira de Freitas, Gabriela Fulco, Andréia Veras Gonçalves, Ricardo Queiroz Gurgel, Juliana Herrero-Silva, Cristina Barroso Hofer, Mariana de Carvalho Leal, Lamin Leigh, Mariana Ramos Pitta Lima, Aline Siqueira Alves Lopes, Tereza Lyra, Celina Maria Turchi Martelli, Valquíria Medeiros, Ana Paula Lopes de Melo, Adriana Suely de Oliveira Melo, Demócrito de Barros Miranda-Filho, Ulisses Ramos Montarroyos, Maria Elisabeth Lopes Moreira, Marisa Marcia Mussi-Pinhata, Jeddson do Rêgo Nascimento, Maurício Nogueira, Danielle MS Oliveira, Consuelo Silva de Oliveira, Enny S Paixao, Saulo Duarte Passos, Loveday Penn-Kekana, Júlia M Pescarini, Maria Helena Pinto, Arnaldo Prata-Barbosa, Amber I Raja, Regina C Ramos, Maria Ângela W Rocha, Laura Cunha Rodrigues, Ana Carolyne de Carvalho Lucena Sá, Nuria Sanchez Clemente, Jhulia dos Santos, Darci Neves dos Santos, Deolinda Maria Felin Scalabrin, Lavínia Schuler-Faccini, Antônio Augusto M da Silva, Paula Fabiana Sobral da Silva, Isadora Cristina de Siqueira, Emanuelle Queiroz dos Santos Tenório de Souza, Wayner Vieira de Souza, Maria da Gloria Teixeira, Marilia Dalva Turchi, Sandra Valongueiro, Rômulo AL de Vasconcelos, Liana O Ventura, Ricardo Arraes de Alencar Ximenes, Ana Laura de Sene Amâncio Zara

**Affiliations:** Health Equity Action Lab, Department of Infectious Disease Epidemiology & International Health, London School of Hygiene & Tropical Medicine, London, UK; Departamento de Medicina Interna, Universidade de Pernambuco, Recife 50100-130, Brazil; Departamento de Medicina Tropical, Universidade Federal de Pernambuco, Recife, Brazil

A new strain of Oropouche virus (OROV), a neglected zoonotic orthobunyavirus primarily transmitted to humans in peri-urban settings by *Culicoides paraensis* biting midges, is rapidly spreading, with autochthonous transmission detected in Bolivia, Brazil, Colombia, Cuba, the Dominican Republic, and Peru. On July 12, 2024, Brazilian authorities notified the Pan American Health Organization (PAHO) of a stillbirth following presumed vertical transmission of OROV, as well as the retrospective identification of four neonates who were microcephalic and tested IgM positive for OROV;^1^ further cases of suspected OROV vertical transmission remain under investigation.

Although these case reports raise concerns, it is important to emphasise that, at this time, the evidence base for understanding whether OROV infections during pregnancy might pose risks to maternal-fetal health remains extremely scarce. A rapid research response is needed to generate data to guide health care and assistance for OROV-exposed communities, to determine how frequent OROV vertical transmission is, and to establish whether there are causal associations between prenatal OROV infections and adverse pregnancy outcomes, such as fetal loss or congenital disorders.

As the rapid research response to the possible vertical transmission of OROV accelerates, we reflect on some of the key lessons learned during nearly a decade of congenital Zika research and make the following recommendations.

First, centring the knowledge and expertise of local researchers, public health practitioners, and affected communities. Although multidisciplinary international research collaborations will undoubtedly be invaluable for addressing the many scientific questions surrounding the re-emergence of OROV and for developing novel control measures, including therapeutics and vaccines, OROV has been circulating and studied in Brazil and other countries of the Amazon Basin for decades. The most effective investigations will develop with the leadership of researchers based in affected countries, who can provide unique insights into the epidemiological, ecological, and social characteristics of the outbreak settings, and in partnership with national ministries of health and regional public health authorities and health workers, who are best positioned to monitor for new cases and unusual clinical presentations, to coordinate public laboratories, and to translate research into real-world public health interventions. Moreover, research on paediatric consequences of congenital infections requires establishing long-term relationships with participating families and will be improved by meaningfully engaging in bidirectional and transparent communication with individuals affected by OROV from study design through to dissemination.^2^

Second, cultivating a research culture of collaboration. The cooperative efforts to facilitate individual participant data sharing and joint analyses between the more than 60 cohort studies of congenital Zika, initiated between 2015 and 2017, have been truly laudable.^3^ However, harmonisation of congenital Zika data has been largely retrospective, resulting in delays for risk estimation.^3,4^ PAHO and the Brazilian Ministry of Health have proactively initiated efforts to design standardised study protocols to facilitate prospective harmonisation across new studies of possible OROV vertical transmission, and it will be important to maintain a culture of collaboration, following the model of the congenital Zika research consortia,^3,4^ as the state of OROV-related knowledge evolves.

Third, strengthening surveillance and establishing case definitions that allow for different levels of diagnostic certainty. The scale of the current OROV epidemic remains uncertain due to limitations related to diagnostics and standardised data collection within disease registries. Furthermore, Oropouche disease typically presents as an acute febrile illness that, in the absence of rapid tests, can be misdiagnosed as dengue. As molecular and serological tests for detecting acute and recent OROV infections become more widely available, it will be important to develop a pragmatic and flexible case definition for prenatal infections that considers both the accuracy and time-relevance of different assays.^5^ Although OROV tests are less susceptible to immunological cross-reactivity than Zika virus, determining the timing of infections during pregnancy will still be challenging due to the brief viraemic window during acute infections and uncertainties regarding the baseline seroprevalence, the immune response kinetics, and the frequency of asymptomatic infections. Prospective studies should consider repeated testing during pregnancy to identify seroconversion events and truly negative OROV-unexposed control individuals.^6^

Fourth, triangulating evidence to investigate causality, define the natural history of disease, and understand socioeconomic consequences. Although OROV has unique characteristics in terms of virology, transmission, and clinical consequences, congenital Zika research provides a roadmap for conducting scientifically rigorous epidemiological investigations of the possible vertical transmission of an arthropod-borne virus. As a starting point for OROV researchers, case–control studies will be valuable to rapidly investigate causality and exclude alternative hypotheses.^7^ Surveillance-based and antenatal care-based pregnancy cohort studies will also be crucial to define vertical transmission rates and adverse pregnancy outcome risks associated with OROV infections across the range of gestational ages.^4,6^ Paediatric case series and cohort studies should also be initiated to describe the spectrum of clinical phenotypes and risks of postnatal consequences observed in children identified to have congenital OROV infections.^8^ If a causal link between prenatal OROV infections and congenital disorders is established, important follow-up studies will include registry-based data linkage studies to investigate rare outcomes, such as death,^9^ and social science studies to amplify the perspectives of affected communities and evaluate the wider socioeconomic impacts.^10^ Nevertheless, we recognise that this research will only be possible with the rapid mobilisation and support of international funding agencies.

The possible vertical transmission of OROV deserves a swift, collaborative, scientifically rigorous, and ethically sound research response. Key findings from these investigations should be communicated to broader society in a timely and accessible manner. Ultimately, the biggest lesson learned from the congenital Zika research experience is that we cannot delay.
